# Automatic Infant Movement Assessment Using Pose-LBP Features and a Cost-Sensitive Subspace kNN Ensemble

**DOI:** 10.3390/bioengineering13050516

**Published:** 2026-04-29

**Authors:** Ali Ari, Pelin Atalan Efkere, Ecem Yıldız Çangur, Kamile Uzun Akkaya, Berna Gurler Ari, Bülent Elbasan, Abdulkadir Sengur, Yan Tian

**Affiliations:** 1 Department of Computer Engineering, Faculty of Technology, Gazi University, Ankara 06654, Turkey; ali.ari@gazi.edu.tr; 2 Department of Physical Therapy and Rehabilitation, Faculty of Health Sciences, Gazi University, Ankara 06654, Turkey; pelinatalan@gazi.edu.tr (P.A.E.); ecemyildizcangur@gazi.edu.tr (E.Y.Ç.); kamileuzunakkaya@gazi.edu.tr (K.U.A.); bulentelbasan@gazi.edu.tr (B.E.); 3 Department of Computer Engineering, Engineering Faculty, Turkish National Defence University, Ankara 06654, Turkey; 4 Electrical and Electronics Engineering, Faculty of Technology, Fırat University, Elazığ 23100, Turkey; ksengur@firat.edu.tr; 5 School of Computer Science, Zhejiang Gongshang University, Hangzhou 310018, China; tianyan@zjgsu.edu.cn

**Keywords:** infant movement analysis, pose local binary pattern, pose estimation, cost-sensitive subspace k-nearest neighbor classifier

## Abstract

**Background/Objectives**: Assessment of infant General Movements (GMs) is essential for early detection of neurological disorders such as cerebral palsy, but current methods depend on expert interpretation. This study proposes an automated and interpretable framework for infant movement classification using pose-based representations from RGB videos. **Methods**: A pose-driven pipeline was developed to extract 2D skeletal key points using a two-stage tracking strategy. Joint coordinates were normalized using the shoulder center and inter-shoulder distance. Videos were segmented into overlapping temporal windows, and each segment was represented using Pose-LBP histograms and motion ratio features. Classification was performed with a cost-sensitive subspace k-nearest neighbor ensemble (CSS-kNN-E). Performance was evaluated using stratified 10-fold cross-validation on a five-class infant movement dataset. **Results**: The proposed method achieved 99.16% (±0.48%) accuracy, 99.19% (±0.50%) sensitivity, 99.76% (±0.13%) specificity, and 99.23% (±0.48%) F1-score. The model demonstrated strong discrimination across classes and robustness to class imbalance. **Conclusions**: The framework provides an accurate and scalable solution for automated infant movement analysis. It reduces dependency on expert evaluation and has strong potential for early clinical screening and decision support.

## 1. Introduction

Spontaneous movements are simple or complex involuntary movements produced by the immature nervous system that occur without sensory input [1,2]. Spontaneous movements are thought to be triggered by central pattern generators (CPGs) located in the spinal cord and brainstem [3]. Sensory stimuli from the periphery and pathways descending from cortical and subcortical structures modulate CPGs [4]. Examples of spontaneous movements in the fetal period include sucking, hiccupping, yawning, startling, stretching, isolated limb movements, and General Movements (GMs). These movements are initially simple and low-amplitude, but become more complex over time; their amplitude, variety, and duration increase [1]. GMs, one type of spontaneous movement, are movements with variety and fluidity that occur from the eighth week of fetal life until approximately the 20th post-term week. The period up to week 10 constitutes the writhing movement period, and the period from week 10 to week 20 constitutes the fidgety movement period [5,6]. The absence of fidgety movements or the presence of abnormal fidgety movements is an important determinant of neurological damage.

Cerebral palsy (CP) is an umbrella term for disorders resulting from damage to the immature brain of newborns, affecting posture, movement, and muscle tone. CP accounts for a significant portion of childhood morbidity [7]. Early diagnosis of CP is important for improving disease prognosis, reducing healthcare costs, and planning relevant strategies [8]. Magnetic resonance imaging (MRI), neurological assessment, and GM analysis are used together in the diagnosis of CP, and this diagnosis is highly accurate (sensitivity: 98%, specificity: 99%) [9]. When GM analysis is used alone, it has a sensitivity of 95% and a specificity of 97% [10]. GM analysis is a method described by Heinz Prechtl. Prechtl suggests that in infants with nervous system disorders, GMs lose their complex and variable character and become less fluid. Therefore, detailed analysis of GMs alone plays a crucial role in diagnosing CP [5].

GM analysis is performed by having specialists who have received training in this method watch 3–5 min video recordings of infants and categorize the infant’s movements according to whether they correspond to the writhing or fidgety stage [5]. This method, which enables the early diagnosis of CP, an important public health problem, should be applied by as many healthcare professionals as possible, as this will lead to the application of necessary early interventions, the alleviation of the disease course, and the reduction in healthcare costs. However, GM analysis training is costly, and only a small number of healthcare professionals have this training. Having training alone does not mean that all analyses can be done correctly. Accurate categorization of movements in videos requires the evaluator’s many years of experience. The limited nature of the human eye and judgment increases the importance of automation in such important decisions.

With the advancements in technology and artificial intelligence applications, various studies are being conducted to automate diagnostic and decision-making processes in many areas of medicine. Automation provides benefits such as speeding up diagnostic and decision-making processes, increasing the rate of correct decisions for a larger number of users, and spreading the use of diagnostic techniques. Studies on the automation of GM analysis have been on the agenda for some time. Although these studies use different methods, the aim is the same: to spread GM analysis, which has a very high predictive power in the diagnosis of cerebral palsy, and to enable early diagnosis for a greater number of infants. Current studies on this subject are summarized below.

In this work, we propose a novel pose-based approach to automate infant movement classification from RGB video. Moving away from either pure reliance on handcrafted motion statistics or heavyweight fully end-to-end deep learning, our contribution fuses a structured pose representation with a lightweight and discriminative classifier. We begin by converting raw video frames into normalized skeletal trajectories using a robust human detection and key point estimation pipeline. These are further segmented into fixed-length, overlapping temporal windows, allowing the model to capture short-term movement dynamics while preserving sequence coherence. For each window, the movement patterns are described by a compact yet expressive set of features derived from how joints relate spatially and how they evolve over time. More precisely, posture is captured with a Pose-LBP encoding the relative configurations of joints, while motion is summarized via activity ratios across joints. This combination produces interpretable features that reflect both static posture and dynamic behavior relevant to clinical infant movement assessment. We apply CSS-kNN-E classifier to handle the class imbalance problem and improve its robustness for any given movement class. The feature subspace sampling, combined with distance-based learning, brings diversity and locality to the classifier while keeping the classification lightweight. Extensive cross-validation results prove that the framework achieves very strong accuracy, sensitivity, specificity, and F1-scores for each of the five classes while outperforming several traditional baselines.

The key contributions are as follows:

We present a complete, interpretable end-to-end pipeline, from raw video input to segment and segment-level movement classification.

We propose a novel pose-based feature representation that jointly captures postural configuration and motion intensity in a compact form.

We apply CSS-kNN-E to infant movement classification, delivering strong performance with simplicity and transparency.

The remainder of this paper is organized as follows. Section 2 describes the materials and methods, including the proposed framework and the related theoretical background. Section 3 introduces the dataset, presents the experimental setup, and reports the obtained results. An ablation study is also included to evaluate the contribution of different components of the proposed model. Section 4 discusses the findings and compares the proposed approach with traditional machine learning methods. Finally, Section 5 concludes the paper and outlines directions for future work.

### Literature Review

Hesse et al. (2018) [11] developed a system to analyze infant movements in a computer environment. In this study, infant movements were recorded using RGB-D cameras and modeled in 3D to create an infant body model, SMIL. Thanks to this developed 3D model, infant movements could be recreated in a computer environment with a very close accuracy to reality. In the evaluations, a high level of agreement was found between the real video recordings and the artificial movement videos created using the SMIL model (90%). These results revealed that the system provides a robust infrastructure for future automated analysis of GMs [11]. This study has shown that, before implementing GMA automation, consistently predicting body positions is crucial to ensuring its reliability. In their study, which did not include GMA classification but instead used spatiotemporal parameters for position estimation, Moccia et al. examined 4000 test frames from NICU videos of 16 infants. The authors proposed a two-stage deep learning model. This model first detected joint movements and, using a regression model trained on these detections, accurately predicted joint movements. The lack of GMA classification in the system, which was successful in prediction, was noted as a limitation [12]. These studies have provided the infrastructure for GMA automation and highlighted the need to reduce labeling requirements.

In their study, Ni et al. [13] aimed to develop a system that could more consistently predict infant body parts and positions with a small number of labels, thereby making GMA predictions more reliable. They showed that when they incorporated the results from their developed system into the GMA prediction processes, the GMA prediction also became more consistent.

Rajpopat et al. [14] aimed to fill the gap in the literature by combining both body part and frequency-time parameters, rather than focusing on them separately. In their study, they integrated a body part-based framework with the existing pose-based model. In the study, movement coordinates were extracted for eight separate joints (ankle, knee, wrist, elbow) using OpenPose, and time and frequency parameters were analyzed together. A distinguishing feature of the study is that instead of generating a model for the entire body, a separate Gradient Boosting Decision Tree (GBDT) was trained for each joint. The extracted models were examined using a rating-based ensemble, achieving an accuracy rate of up to 98.94%. The study’s weaknesses include limited public datasets (MINI RGBD and RVI-38) and the categorization of fidgety movements as present/absent. The study lacks labeling for other GMA subgroups.

In another study using the MINI RGBD and RVI-38 databases, pose estimation was performed on 2B and 3B RGB videos of infants using different methods such as MediaPipe, OpenPose, and MeTRAbs. From the extracted poses, the distances and positions of the joints relative to each other, as well as kinematic characteristics of the movement, such as speed, acceleration, and symmetry, were examined. Based on these characteristics and in parallel with GMA, an attempt was made to classify the movement as normal or as an infant movement that posed a risk of cerebral palsy (CP). The developed method achieved accuracy rates of 92% and 93.37%, but the limited dataset was noted as a limitation [15].

In their study, Pellano et al. [16] developed a velocity- and angle-based perturbation framework to assess the biomechanical consistency of Graph Convolutional Networks, an ensemble model previously developed to predict CP. They found that velocity perturbations were more effective than angle perturbations in the model’s decision-making mechanism, in parallel with GMA analysis. Considering that in GMA analysis, the speed and quality of motion are more significant than position, they determined that the initially trained model was based on biomechanically consistent decision-making mechanisms.

Kulvicius et al. [17] reported that studies proposing the automation of GMA achieved success rates of around 90%, but they suggested using multiple sensors (visual, inertial, and pressure) and a sensor fusion approach to surpass this rate. They proposed that different sensors could capture points missed by others in detecting the same movement. They analyzed data collected from 51 infants using wearable sensors, a pressure mat, and an RGB camera during GMA recordings using convolutional neural network (CNN)-based classifiers. They compared the success of two approaches, combining features into a single network (which they called early fusion) and combining outputs from separate networks (which they called late fusion), and found that late fusion was more successful. The balanced accuracy rate was 94.5% when video recordings, wearable sensor data, and pressure mat data were combined. Despite the high accuracy rate, the study’s limitations include the need for data collection in a clinical setting and the requirement for multiple recordings.

Groos et al. [18] developed a deep learning-based system aimed at predicting the risk of CP in 9–18-week-old infants using GMA videos. Movement data from 19 selected anatomical points were converted into temporal skeletal sequences. These sequences were analyzed in 5 s segments by an ensemble of 70 artificial experts. The system achieved a 90.6% accuracy rate, distinguishing not only the risk of CP but also its subtypes. This was noted as a strong point of the system.

Passmore et al. [19] proposed a deep learning framework using 503 home video recordings from caregivers of 341 infants with corrected ages ranging from 12 to 18 weeks. In this model, DeepLabCut, a position-prediction model, was trained to automatically predict 18 manually marked points on infants’ bodies. The highest accuracy was obtained for the eyes, and the lowest for the hips. Positional data were preprocessed using a specialized pipeline to standardize the video recordings before classification. The videos were divided into 5 s segments, and a convolutional neural network was trained to differentiate between abnormal/absent fidgety and normal fidgety. In this study, the authors achieved 76% sensitivity and 94% negative predictive value in predicting GM assessment with their proposed method. The study’s strength is the use of a database of videos recorded by caregivers at home. The weakness in this study is the lack of an attempt to predict other GMA subgroups.

Unlike other studies, Letzkus et al. [20] focused on predicting earlier-onset writhing movements in infants using machine learning. A model trained on 85 videos recorded in a NICU environment automatically identified 17 key points in the infants. Then, the joint angles between these points and their changes over time were determined using cosine similarity. The resulting autocorrelation data were used to distinguish whether the videos belonged to the Cramped-Synchronized or Normal Writhing category. Due to the small number of data points and the lack of a classifier in the Cramped-Synchronized category, the accuracy rate was not reported; however, the study highlighted an important point for early diagnosis of cerebral palsy (CP) by using GMA analysis at an earlier stage than the fidgety period.

Soualmi et al. [21] aimed to predict body poses in infant videos through deep learning using their AGMA-PESS software, and to select the sequence with the most intense movement in the video. By comparing the selections of the developed system with the selections of expert evaluators, it was shown that the vast majority of the selected sequences coincided with the selection of at least one expert. The results of the study revealed that the AGMA-PESS approach saves time compared to the manual GM analysis process and can offer a reliable automated sequence selection and pose prediction solution.

Unlike other studies, Gao et al. [22] quantitatively evaluated the number/intensity of fidgety movements, rather than their presence or absence. They aimed to automate GMA using a deep learning-based motor assessment model (MAM). By extracting joint coordinates from videos using 3D pose estimation, creating inter-joint distance matrices, and analyzing these representations with a transformer-based model, the authors achieved an accuracy rate of 93–94%. The strength of the study lies in achieving a high accuracy rate using a single camera without the need for sensor fusion.

Although current literature shows significant advancements in GMA automation and high accuracy rates, most approaches rely on additional hardware requirements or limited generalizability. Furthermore, studies are limited in their coverage of both writhing and fidgety phases. This study proposes a deep learning model that aims to automatically and holistically classify all phases of GMA movements using only video data.

## 2. Materials and Methods

**Figure 1:**
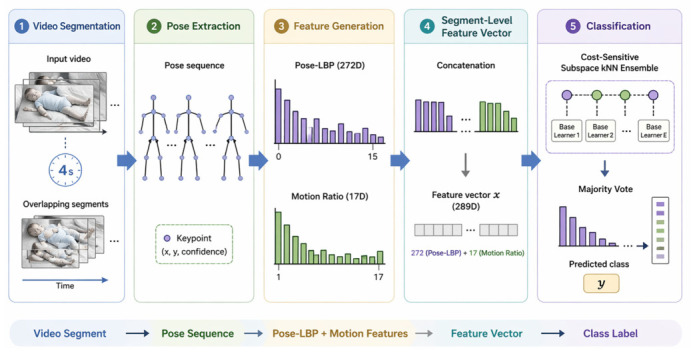
The proposed Pose-LBP-based infant movement detection system.

The framework for infant movement classification using Pose-LBP is shown in Figure 1. From the illustrated figure, the framework uses a pipeline approach starting with the parsing of a video clip of a duration of four seconds. From this clip, a sequence of poses is derived through the detection of key skeletal points. The skeletal points are then converted into Pose-LBP features. The features are represented as a histogram of Pose-LBP features of a total of 272 features along with a motion ratio of a total of 17 features. In addition, Pose-LBP features and motion-based features are extracted and concatenated to the Pose-LBP features to construct a final feature vector. This forms a feature vector of a total of 289 features. The feature vector is then used as input for a cost-sensitive subspace kNN ensemble classification framework. The framework uses a majority voting scheme for the determination of the final movement class. The proposed system was implemented using MATLAB (version R2023a, MathWorks, Natick, MA, USA).

### 2.1. Pose Time Series Representation

Let a video sequence depict spontaneous infant movements captured under unconstrained conditions [23]. The main idea here is to analyze the patterns of movement using skeleton representations as opposed to intensity values of pixels. Skeleton representations are a compact and meaningful way of describing the pose of a body and are quite robust to illumination changes, presence of clutter in the background, and variability in the body appearances. However, the joint locations are not directly suitable for the task of classification as they are very sensitive to camera placement, body size, and variability from one subject to another. Thus, the aim of the proposed approach is to design a pose-normalized, local, and relative motion representation that takes a cue from the idea of the LBP operator but applies it to a skeleton structure. This representation is termed as Pose-LBP.

Each video is partitioned into fixed-length temporal segments of duration $T_{s}$ seconds. Given a sampling interval Δt, each segment consists of $T=\left\lfloor \frac{T_{s}}{\Delta t}\right\rfloor$ frames. For each frame t∈{1,…,T}, a pose estimation algorithm extracts a set of 17 body joints. Each joint is represented by a 2D coordinate:

$$P_{t,j}={\left(x_{t,j},y_{t,j}\right)}^{T}\in R^{2},j=1,2,\ldots ,17.$$

Stacking all joints across time yields the pose tensor:

$$P=\left\{p_{t,j}\right\}\in R^{T\times 17\times 2},$$

This representation captures the full spatiotemporal evolution of the skeletal structure within a segment.

### 2.2. Pose Normalization

To eliminate global translation effects, each frame is centered with respect to a torso reference point [24]. Let *LS* and *RS* denote the left and right shoulder joints. The torso center at time *t* is defined as

$$c_{t}=\frac{p_{t,LS}+p_{t,RS}}{2}$$

All joint coordinates are translated accordingly:

$$p_{t,j}^{\prime }=p_{t,j}-c_{t}$$

Inter-subject size variations and camera zoom effects are addressed by normalizing with respect to shoulder width:

$$s_{t}={\left\|p_{t,LS}-p_{t,RS}\right\|}_{2}$$

The final normalized joint coordinates are given by

$${\tilde{p}}_{t,j}=\{\begin{array}{c} \frac{p_{t,j}^{\prime }}{s_{t}},\;\;\;s_{t}>0\;and\;joint\;j\;is\;valid,\\ 0,\;\;\;\;\;\;\;\;\;\;\;\;\;\;\;\;\;\;\;\;\;\;\;\;\;\;\;\;\;\;\;\;\;\;\;\;otherwise \end{array}$$

This normalization ensures translation and scale invariance, a critical requirement for comparing movement patterns across subjects and recordings.

### 2.3. Skeleton Graph and Local Neighborhoods

The skeleton is modeled as a graph *G* = (*V*, *E*), where *V* = {1, …, 17} represents joints, and *E* represents anatomical connections [25]. For each joint *j* ∈ *V*, a local neighborhood *N*(*j*) ⊂ *V* is defined according to anatomical adjacency. To limit complexity and noise sensitivity, at most, four neighbors are retained:

|N(j)|≤4

This graph structure allows Pose-LBP to operate on anatomically meaningful local regions, analogous to pixel neighborhoods in classical LBP. For a given joint *j*, neighbor *k* ∈ *N*(*j*), and time *t*, the Euclidean distance between normalized joints is

$$d_{t}^{\left(j,k\right)}={\left\|{\tilde{p}}_{t,j}-{\tilde{p}}_{t,k}\right\|}_{2}$$

Rather than comparing distances across frames directly, a segment-adaptive baseline is introduced. The reference distance for the joint–neighbor pair (*j*, *k*) is computed as

$${\bar{d}}^{\left(j,k\right)}=\frac{1}{T}\sum_{t=1}^{T}d_{t}^{\left(j,k\right)}$$

This reference captures the typical spatial relationship between joint *j* and neighbor *k* within the segment.

### 2.4. Angular Encoding of Local Articulation

Distance information alone may not capture changes in joint articulation direction [26]. Therefore, an angular component is introduced when at least two neighbors are available. Let *k*_1_, *k*_2_ ∈ *N*(*j*) be the first two neighbors. Define the vectors

$${v_{t,1}=\tilde{p}}_{t,k_{1}}-{\tilde{p}}_{t,j},\;\;\;\;\;\;\;{v_{t,2}=\tilde{p}}_{t,k_{2}}-{\tilde{p}}_{t,j}$$

The angle between them is calculated as

$$\theta_{t,j}=\cos^{-1}\left(\frac{v_{t,1}v_{t,2}}{{\left\|v_{t,1}\right\|}_{2}{\left\|v_{t,2}\right\|}_{2}+\varepsilon }\right)$$

where ε is a small enough parameter. A binary angular event is defined as

$$A_{t,j}=I(\theta_{t,j}>\frac{\pi }{12})$$

where I(.) is the indicator function.

### 2.5. Pose-LBP Code Construction

For each joint *j* and time *t*, binary bits are assigned for each neighbor index *b*:

$$B_{t}\left(j,b\right)=\{\begin{array}{c} B_{t}^{dist}\left(j,n_{j,1}\right)\vee A_{t,j},\;\;\;b=1\\ B_{t}^{dist}\left(j,n_{j,b}\right),\;\;\;\;\;\;\;\;\;\;\;\;\;\;b>1 \end{array}$$

where $B_{t}^{dist}(j,k)$ shows the binary distance. The binary distance is calculated as

$$B_{t}^{dist}\left(j,k\right)=\{\begin{array}{c} 1,\;\;\;\;d_{t}^{\left(j,k\right)}>{\bar{d}}^{\left(j,k\right)}\;\\ 0,\;\;\;\;\;\;\;\;\;\;\;otherwise \end{array}$$

This binary distance encodes whether the joint is more extended or more contracted than usual with respect to its neighbor. Thus, the Pose-LBP code is then obtained by binary weighting:

$$c_{t,j}=\sum_{b=1}^{\left|N(j)\right|}B_{t}(i,b)2^{b-1}$$

where $c_{t,j}\in \{0,1,\ldots ,15\}$. Collectively, the Pose-LBP codes form a matrix $C\in {\left\{0,\ldots ,15\right\}}^{T\times 17}$. Each element $c_{t,j}$ compactly represents the local skeletal configuration around joint *j* at time *t*.

To obtain a fixed-length representation independent of temporal alignment, Pose-LBP codes are aggregated over time using histograms. For joint *j*, a normalized histogram is computed:

$$h_{j}\left(m\right)=\frac{1}{T}\sum_{t=1}^{T}I\left(c_{t,j}=m\right),m=0,\ldots ,15$$

Concatenating histograms for all joints yields

$$h\in R^{17\times 16}\equiv R^{272}$$

This representation captures the distribution of local pose patterns over the segment.

### 2.6. Motion-Based Features

To explicitly encode movement intensity, a per-joint motion ratio is added [27]. The velocity magnitude is defined as

$$v_{t,j}={\left\|{\tilde{p}}_{t+1,j}-{\tilde{p}}_{t,j}\right\|}_{2}$$

An adaptive threshold is computed:

$$\tau_{v}=max(5\times 10^{-3},{percentile}_{70}\left\{v_{t,j}\right\})$$

The motion ratio for joint *j* is

$$r_{j}=\frac{1}{T-1}\sum_{t=1}^{T}I(v_{t,j}>\tau_{v})$$

This produces $r\in R^{17}$. The final feature vector is obtained by concatenation of the *h* and *r* such as $f=[h,r]\in R^{289}$.

### 2.7. Cost-Sensitive Subspace kNN Ensemble (CSS-kNN-E)

The proposed classifier is a random subspace ensemble whose base learner is a 1-nearest neighbor (1-NN) classifier computed in a randomly selected feature subspace [28]. Let *x* ∈ *RD* denote a standardized feature vector and *y* ∈ {1,…,*C*} its class label [29]. In each ensemble iteration *m* = 1,…,*M* a subset of feature indices *F_m_* ⊂ {1,…,*D*} is sampled uniformly at random with fixed cardinality ∣*F_m_*∣ = *N*pred. The *m*-th base classifier *h_m_*(⋅) operates only on the selected subspace *F_m_*. Given a test sample *x*, the distance to a training sample *x_i_* within subspace *F_m_* is computed using the cityblock (Manhattan) distance:

$$d_{m}\left(x,x_{i}\right)=\sum_{d\in F_{m}}\left|x_{d}-x_{i,d}\right|$$

The 1-NN base prediction is then defined as

$$h_{m}\left(x\right)=y_{i^{\backslash *}},i^{\backslash *}=\arg\;{min}_{i}(d_{m}(x,x_{i}))$$

Thus, each learner provides a class label by selecting the closest training sample in its own randomly chosen feature subspace, which promotes classifier diversity and reduces sensitivity to redundant or noisy dimensions. The ensemble combines the *M* base predictions through majority voting. The final predicted class is

$$\hat{y}\left(x\right)=\arg\underset{c\in \{1,\ldots ,C\}}{\max}\sum_{m=1}^{M}g(h_{m}\left(x\right)=c)$$

where g(.) is a function indicator. To address class imbalance, the training is performed with a cost matrix that penalizes errors on minority classes more heavily. Let *f_i_* be the empirical frequency of class *i*

$$f_{i}=\frac{n_{i}}{\sum_{k=1}^{C}n_{k}}$$

where *n_i_* is the number of training samples in class *i*. The misclassification cost matrix *C* ∈ *RC*×*C* is defined as

$$C_{i,j}=\{\begin{array}{c} 0,\;\;\;\;\;i=j\\ \frac{1}{f_{i}},\;\;\;i\neq j \end{array}$$

This cost-sensitive formulation encourages the ensemble to reduce false negatives for rare classes by increasing the loss associated with misclassifying samples from those classes.

## 3. Experimental Works and Results

### 3.1. Dataset

In this paper, a new BERA-GAZI-RGB dataset was introduced. The dataset was collected in the Developmental Physiotherapy and Pediatric Rehabilitation Unit in Gazi University’s Faculty of Health Sciences. These video recordings were collected out between 14 January 2025 and 14 December 2025, for post-term infants between 0 and 2 months and 2–5 months. The collected new dataset includes 400 video samples obtained from 200 high-risk infant recordings, all of which were expertly evaluated by trained Pediatric Physiotherapists in General Movement Analysis techniques. These analyses include Normal Writhing, Poor Repertoire, Cramp-Synchronized, Fidgety Present, Fidgety Absent Movement analyses. To ensure consistency among samples, 20 s cuts were taken from the original footage. As a new proposed dataset in this research paper, 400 samples of video data were taken, which are evenly distributed among the five movement analyses, which include a massive range of Normal and Abnormal General Infant Movement patterns.

### 3.2. Results

All experiments were implemented in MATLAB (version R2023a) on a workstation equipped with an NVIDIA GeForce RTX 3090 GPU and 24 GB of RAM. Video recordings were divided into fixed-length overlapping temporal segments. Each segment was 4 s long, frames were sampled every 0.05 s (approximately 20 fps), and consecutive segments overlapped by 0.5 s, resulting in 80 frames per segment. Across the complete dataset, 78,305 frames were processed during pose extraction. Among these, 468 frames did not yield valid pose detections and were therefore represented by zero-valued vectors, corresponding to an invalid frame rate of 0.60%. This strategy preserved temporal continuity within fixed-length segments while avoiding the exclusion of partially usable video recordings.

For each sampled frame, a two-stage pose extraction pipeline was applied. First, a person detector localized the infant in the frame, selecting the bounding box with the highest confidence when multiple detections were present. Second, an HRNet-based key point detector estimated 17 two-dimensional body joints within the detected region. Frames without valid detections were represented by zero vectors to preserve temporal consistency. The extracted joint coordinates were normalized to achieve translation and scale invariance by centering the skeleton between the left and right shoulders and scaling it by the inter-shoulder distance. Each frame was therefore represented as a 34-dimensional pose vector containing the x and y coordinates of the 17 joints. Segment-level features were constructed using a Pose-based LBP representation. For each joint, a predefined skeletal neighborhood was used to define anatomically related joints. At each frame, binary comparisons based on relative joint distances were performed between a joint and its neighbors. An additional angle-based binary condition was computed using the two closest neighbors. These binary decisions were combined into a four-bit Pose-LBP code ranging from 0 to 15 for each joint at each frame. Pose-LBP codes were aggregated over time using normalized 16-bin histograms for each joint, producing a 272-dimensional feature vector. To incorporate motion intensity, joint velocities were computed and converted into motion ratio features representing the proportion of frames with significant movement for each joint. The final segment representation was obtained by concatenating the Pose-LBP histogram and motion ratio features into a 289-dimensional vector. All features were standardized using z-score normalization. Classification was performed using a cost-sensitive subspace k-nearest neighbor ensemble, where multiple 1-NN classifiers were trained on randomly selected feature subsets and combined via majority voting. Class imbalance was addressed by assigning higher misclassification costs to underrepresented classes. Performance was evaluated using ten-fold cross-validation with segment-level data partitioning to avoid subject leakage.

**Figure 2:**
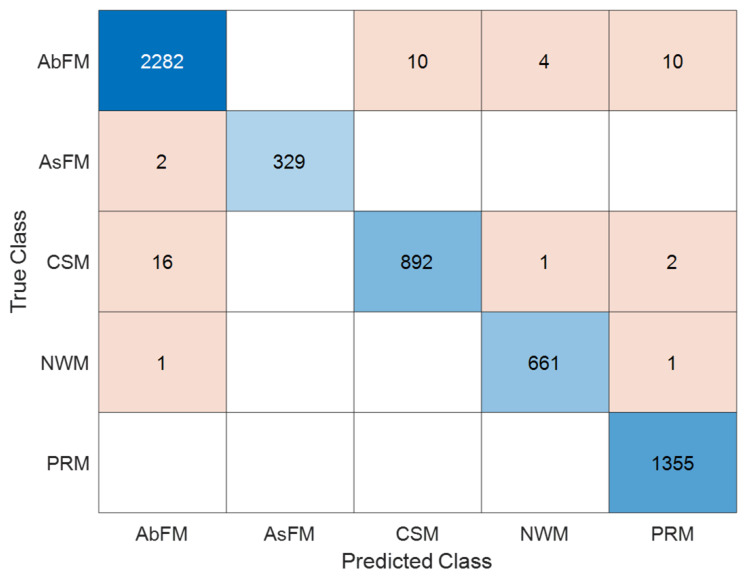
Confusion matrix of the CSS-kNN-E classifier showing true versus predicted classes under 10-fold cross-validation.

We used a 10-fold cross-validation protocol to figure out the performance evaluation metrics, which are accuracy, sensitivity, specificity, and F1-score [30]. In this case, the dataset was split into ten random parts. In every iteration, nine folds were used for training and one fold was used for testing. The final results were the average of all the folds. We tested a number of classifiers with fixed parameter settings that were chosen to make sure that all experiments were stable, fair, and could be repeated. Figure 2 illustrates the confusion matrix and class-based evaluation values for the baseline kNN method for the five movement classes: Abnormal Fidgety Movements (AbFMs), Absent Fidgety Movements (AsFMs), Cramped-Synchronized Movements (CSMs), Normal Writhing Movements (NWMs), and Poor Repertoire Movements (PRMs).

Figure 2 shows the confusion matrix for the CSS-kNN-E classifier on 10-fold cross-validation. With 5566 samples in total, the system matches the classification with high precision as reflected in the strong diagonal of the confusion matrix. In particular, it correctly classifies 2282 samples of AbFM, 329 of AsFM, 892 of CSM, 661 of NWM, and 1355 of PRM. Incorrect classification occurs sparingly and mostly among movement types that are medically equivalent. In particular, 24 samples of AbFM are incorrectly classified to other classes, with 19 samples of CSM incorrectly classified—mostly to AbFM and PRM. Normal Writhing Movements and Poor Repertoire Movements are nearly perfectly classified with no errors and with only two errors, respectively. In summary, the proposed approach is strong on its discriminative capability with good tolerance on inter-class similarities.

Table 1 summarizes the performance of the CSS-kNN-E classifier over folds in a setup of 10-fold cross-validation. Accuracy is consistently high in every fold, clustering around an average of 0.9916, indicating strong generalization and low sensitivity to the way the train and test splits are conducted. Sensitivity remains above 0.98 for all folds, indicating that movement abnormalities and normal patterns are reliably detected—a crucial aspect for dependable clinical assessment. Specificity is very high, averaging 0.9976, reflecting very few false positives and clear separation between the classes of movement. This is reinforced by the F1-score, which balances precision and recall, with a mean of 0.9923. In addition, the small standard deviation for all the metrics shows robustness and stability, emphasizing the readiness of the method for practical use in automated infant movement analysis systems.

**Figure 3:**
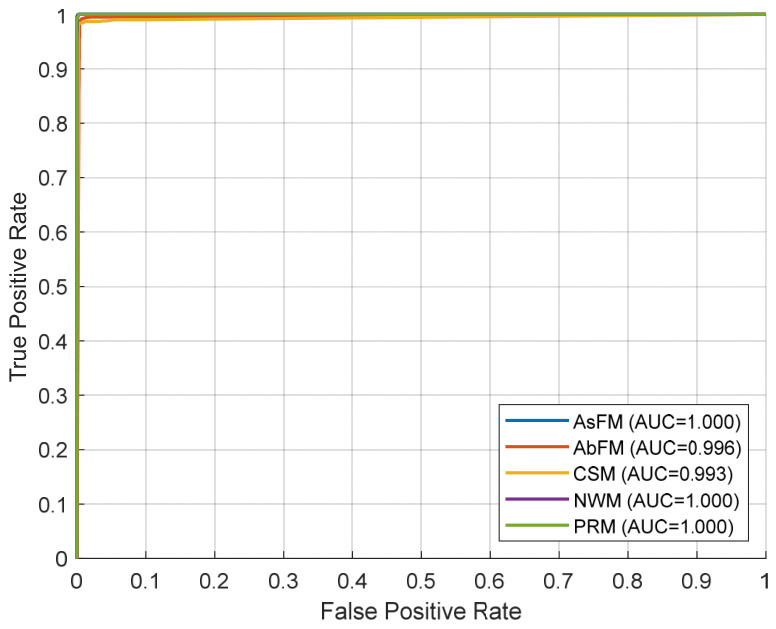
Obtained ROC curves (one-vs-rest, out-of-fold) for each class.

The ROC curves in Figure 3 illustrate how well each class of movement can be distinguished from all other classes in a one vs. all classification problem, based on out-of-fold predictions of the CSS-KNN-E classifier with 10-fold cross-validation on the entire dataset. The ROC plots are a graphical illustration of how different true and false positive rates are traded off, and a particular class of movement achieves an Area Under the ROC Curve (AUC) of very nearly 1.0 in each case, indicating near-perfect separation of each class from all others. The classes AsFM, NWM, and PRM are of course perfectly separable from each other, with actual AUC values of 1.000, and AbFM and CSM are very closely separable with AUC values of 0.996 and 0.993, respectively. The steep rise in each ROC curve near the origin of the plot indicates that each class of movement can be identified with very high sensitivity at very low false positive rates, indicating that each class of movement is very well represented in feature space and that the cost-sensitive subspace kNN ensemble classifier is very effective at overcoming class imbalance and class similarities.

**Figure 4:**
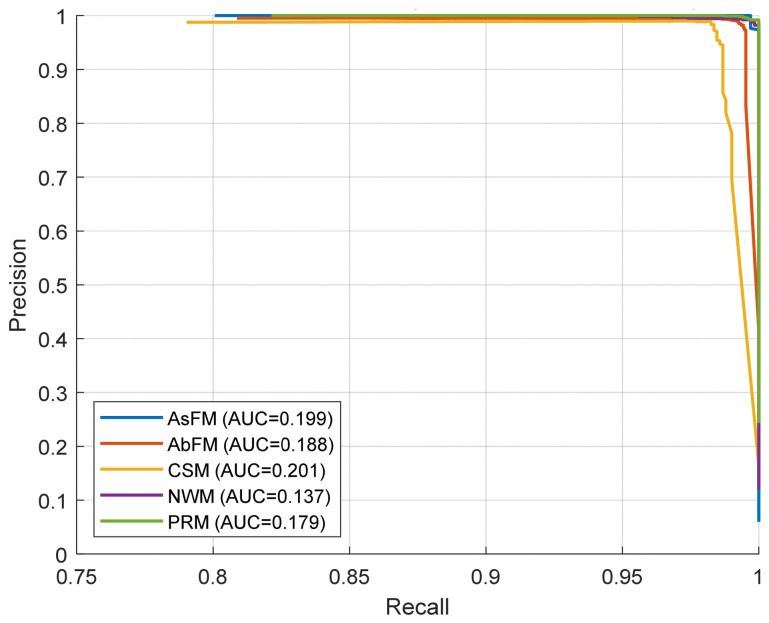
Obtained precision–recall curves (one-vs-rest, out-of-fold) for each class.

Figure 4 illustrates the precision–recall curves for each class together with their corresponding PR-AUC values. Although the PR-AUC values appear numerically low, this should be interpreted in the context of the one-vs-rest setting and class prevalence. In precision–recall analysis, the no-skill baseline corresponds to the prevalence of the positive class. Since each class is evaluated against all remaining classes, minority classes inherently yield low baseline PR-AUC values. Therefore, PR-AUC values in the range of 0.137–0.201 should be interpreted relative to their respective class prevalence rather than in absolute terms. By contrast, ROC-AUC is less sensitive to class imbalance and may remain high even when PR-AUC appears modest. This explains why strong ROC-AUC values can coexist with comparatively low PR-AUC values in imbalanced multi-class problems. In our results, the relatively lower PR-AUC values for classes such as AbFM and CSM may also reflect partial overlap between clinically similar movement patterns, while the lower value for NWM is likely influenced by its smaller representation and similarity to some abnormal movement categories.

### 3.3. Ablation Study

An ablation study is performed to examine in detail the contribution of each component of our CSS-kNN-E approach [31]. The baseline corresponds to our complete approach, which combines Pose-LBP histogram features with those derived from the motion ratio, using cost-sensitive learning and a kNN ensemble in a transformed space. To examine the importance of using cost-sensitive learning, our approach without the misclassification cost matrix is evaluated. To analyze feature combination, two specific approaches, corresponding to using only Pose-LBP histogram features and using only the motion-based features, with cost-sensitive subspace kNN learning, respectively, are considered. To evaluate the contribution of the ensemble itself, we replaced the subspace ensemble with a single kNN classifier without altering the combined Pose-LBP and motion feature set and cost sensitivity. Further experiments validated robustness against significant hyper-parameters by modifying the distance measure to Euclidean distance, incrementing the size of the search neighborhood to three, and reducing the number of cycles of ensembles for learning to 100. All variants were tested under the same ten-fold cross-validation scenario, and the results are presented as the average and standard deviation for accuracy, macro-sensitivity, macro-specificity, and macro F1-score. The study helps to understand the extent to which each component, from feature engineering, cost sensitivity, ensemble method, to hyper-parameter, impacts the solution.

Table 2 presents the impact of the ablation test on the performance of the CSS-kNN-E classifier when using 10-fold cross-validation as a metric measure of accuracy, sensitivity, specificity, and macro F1-score. Clearly, the best performance measure with an accuracy of 0.9916 ± 0.0048 and a macro F1-score of 0.9923 ± 0.0048 is presented by the combination of the PoseHist and motion, cost-sensitive, subspace kNN ensemble. This clearly shows how critical it is to have the spatial patterns of pose as well as motion features towards gaining effective results of classifying movements. Removing the cost-sensitive component has little effect on the performance and slightly increases the accuracy to 0.9919 ± 0.0045 and the F1-score to 0.9926 ± 0.0045, which indicates that the ensemble model design is functioning to counter the imbalance in the datasets. Using only the PoseHist feature results in an accuracy of 0.9777 ± 0.0072 and an F1-score of 0.9777 ± 0.0085, which means the static pose feature alone is insufficient for the classification of movements. The degradation is more prominent while working with just motion features. Accuracy degrades to 0.9373 ± 0.0136 and F1-score to 0.9394 ± 0.0141. This clearly showcases the significance of the spatial relationships between poses involved in the Pose-LBP histogram features. Replacing the ensemble with a single kNN classifier slightly improves the degradation of all measures; this clearly verifies the effectiveness of diversity brought by the ensemble. Using cityblock versus Euclidean distance worsens the performance because accuracy reduces to 0.9874 ± 0.0056, so cityblock is better at representing the distribution of the features. Using a larger size of the neighborhood by setting k to three worsens the performance, while reducing the number of ensemble passes to 100 provides a similar outcome to running the full model.

As observed in Table 2, removing the cost-sensitive component leads to a very slight increase in overall accuracy and F1-score. This suggests that, in the current dataset, the main performance gain is primarily driven by the ensemble formulation and the discriminative power of the combined PoseHist and motion features, rather than by the cost matrix itself. In other words, the random subspace kNN ensemble already provides substantial robustness against class imbalance through classifier diversity and feature subspace sampling. Nevertheless, the cost-sensitive formulation was retained as a principled mechanism to emphasize minority classes and reduce the risk of false negatives in clinically important but less represented movement categories. Therefore, while its quantitative contribution is limited in the present experiments, it remains a meaningful design choice from a class imbalance perspective.

## 4. Discussions

In this study, we propose a novel pose-based classification framework for automatic infant movement assessment that integrates skeleton-aware local pattern encoding with a cost-sensitive subspace k-nearest neighbor ensemble classifier. The proposed pipeline operates directly on raw video recordings and first extracts two-dimensional skeletal joint trajectories using a person detector followed by an HRNet-based key point estimator [32]. Each video is divided into fixed-length overlapping segments of four seconds, and each segment is represented as a temporal sequence of seventeen body joints. After scale and translation normalization, these joint trajectories are transformed into a compact and discriminative representation using a Pose-LBP encoding scheme that captures local spatial relationships between anatomically connected joints. For each joint, binary codes are generated by comparing inter-joint distances and joint angles against segment-level reference statistics, resulting in frame-wise pose patterns that reflect subtle posture variations. These Pose-LBP codes are aggregated over time using joint-wise histograms and are combined with motion ratio descriptors, yielding a 289-dimensional segment-level feature vector. Classification is performed using a CSS-KNN-E approach, where multiple low-dimensional subspaces are randomly sampled and classified using a nearest neighbor rule with cityblock distance and k equal to one. Class imbalance is explicitly handled through an inverse-frequency cost matrix, ensuring that minority movement classes are not dominated by more frequent patterns [33]. Model evaluation is conducted under ten-fold cross-validation with fold-wise standardization and out-of-fold prediction aggregation. The proposed method achieves a mean classification accuracy of 0.9916 ± 0.0046, an averaged sensitivity of 0.9919 ± 0.0049, specificity of 0.9976 ± 0.0013, and an F1-score of 0.9923 ± 0.0047 across the five movement classes. Compared to ablated variants, the full configuration consistently outperforms models that exclude motion descriptors, remove cost sensitivity, or replace the ensemble with a single kNN classifier. Using Pose-LBP features alone reduces accuracy to 0.9777 ± 0.0072, while motion-only representations lead to a further drop to 0.9373 ± 0.0136, highlighting the complementary nature of spatial pose structure and temporal dynamics. Replacing the cityblock distance with Euclidean distance results in a decrease in accuracy to 0.9874 ± 0.0056, and increasing the neighborhood size to k = 3 yields an accuracy of 0.9867 ± 0.0068. Reducing the number of ensemble cycles to one hundred has a negligible effect on performance, with an accuracy of 0.9917 ± 0.0050, indicating robustness to ensemble size selection.

The performance of various classical machine learning classifiers on ten-fold cross-validation is presented in Table 3. Regarding the simple classifiers, the Bagged Tree classifiers perform best overall with an accuracy of 0.8690 ± 0.0129 and an F1-score of 0.8528 ± 0.0139, outperforming the other simple classifiers in both sensitivity and specificity. The neural network ranks second, performing with an accuracy of 0.7276 ± 0.0214 and an F1-score of 0.7014 ± 0.0276, indicating moderate discriminatory capabilities but lower robustness compared to the ensemble classifiers. The Decision Tree and Linear SVM have similar accuracy values—0.6286 ± 0.0231 and 0.6105 ± 0.0221, respectively—with high specificity values over 0.88 but low sensitivity, indicating difficulty in correctly classifying all the movements. The RBF-SVM classifier fares the worst in the current multi-class classification setting, with an accuracy of merely 0.4127 ± 0.0016 and sensitivity of 0.1992 ± 0.0006, indicating that the classes are inadequately discriminated, although the classifier exhibits a moderate relative importance-based F1-score improvement due to the class imbalance. The Naive Bayes and RUSBoost classifiers perform worst in the overall ranking, with low accuracies below 0.49 and high variability in the sensitive classification rate, indicating the classifiers’ unsuitability for the complex, pose-based feature space captured in the study. Inferring from the results presented in Table 3, the tree-based ensemble classifiers perform as a strong baseline model, but the simple classifiers perform inadequately in grasping the complex pose-based features delineating infant movement patterns, thus necessitating the CSS-kNN-E approach developed in the current work. To complement the conventional machine learning methods, two deep learning architectures, namely a one-dimensional convolutional neural network (1D-CNN) and a long short-term memory network (LSTM), were also evaluated. The 1D-CNN was designed to capture local feature patterns through convolutional operations, whereas the LSTM was employed to model sequential dependencies across the feature representation. In terms of performance, the LSTM consistently outperformed the 1D-CNN across all evaluation metrics. Specifically, the 1D-CNN achieved an accuracy of 0.8834 ± 0.0382, sensitivity of 0.8603 ± 0.0488, specificity of 0.8659 ± 0.0538, and F1-score of 0.8618 ± 0.0507. In comparison, the LSTM yielded higher results, with an accuracy of 0.9397 ± 0.0130, sensitivity of 0.9386 ± 0.0119, specificity of 0.9341 ± 0.0162, and F1-score of 0.9369 ± 0.0146, indicating a more robust and stable classification performance.

We further carried out experiments on the RVI-38 and MINI-RGBD datasets to assess the applicability of the proposed framework on external infant movement datasets. Both datasets were processed using the same pipeline as the main dataset, including pose-based representation, temporal segmentation, Pose-LBP and motion feature extraction, and classification with the proposed model. The obtained results were then reported to provide an additional comparison and to demonstrate the behavior of the method across different datasets. Table 4 shows the obtained results.

Table 4 presents the comparative performance of the proposed Pose-LBP + CSS-kNN ensemble on the RVI-38 and MINI-RGBD datasets. On RVI-38, the proposed method achieved 98.06% accuracy, outperforming McCay et al. [34] with 97.37%, Nguyen-Thai et al. [35] with 81.58%, Sakkos et al. [36] with 84.21%, and Adde et al. [37] with 86.84%. On MINI-RGBD, the proposed method achieved 99.85% accuracy, which is very close to the 100% accuracy reported by McCay et al. [34] and Sakkos et al. [36], and higher than Nguyen-Thai et al. [35] with 91.67% and Adde et al. [37] with 75.00%. These results show that the proposed method performs competitively on both datasets.

The advantages of this study are clear:

1. We introduce the skeleton-aware Pose-LBP representation, which explicitly models how the anatomically connected joints relate to each other in space. This lets us pick up on subtle posture changes that global motion descriptors tend to miss.
2. Combining the Pose-LBP histograms, which capture pose structure with motion ratio features that track temporal dynamics, this approach affords a complementary view that can help better distinguish movement classes similar in joint motion patterns.
3. A CSS-kNN-E classifier was used in order to deal with the problem of class imbalance. It combines inverse-frequency weighting and ensemble diversity to achieve stable performance over both common and rare movements.
4. The use of a subspace ensemble means that the classifier works on several low-dimensional feature subsets, which reduces the sensitivity to noisy or redundant features while still maintaining classification robust.

And the limitations of our study include:

1. Indeed, this approach relies heavily on the precision of skeletal key point detection. Mistakes in pose estimation regarding the location of joints can cascade into the encoding via Pose-LBP and undermine the reliability of the features and the final classification.
2. Due to the multi-step process involved in this system, such as pose extraction, computation for Pose-LBP features, and ensemble-based classification, it enhances computational complexity and requires careful tuning of parameters. Real-time deployment of this approach is hence not quite feasible considering the end-to-end learning models.

## 5. Conclusions

This study proposes an automated framework for assessing infant movement, based on pose-derived representations and a CSS-kNN ensemble classifier. The proposed method transforms raw video into normalized skeletal pose sequences and encodes local spatial relations with a Pose-LBP representation, making objective, repeatable analyses of complex infant motions possible. The resulting features are compact, yet extremely powerful for discriminating various movement patterns by combining detailed postural structure with temporal motion cues. A CSS-kNN ensemble is more robust to class imbalance and has complementary classifiers. Accordingly, the results of ten-fold cross-validation show that this approach consistently outperforms traditional machine learning methods in several metrics, thus proving its efficiency in distinguishing clinically meaningful categories of movement.

Future work will focus on extending the proposed framework to larger and more diverse datasets, including recordings collected under different environmental conditions and from broader clinical populations, in order to further assess generalization capability [38]. An enrichment of the feature set by adding more temporal and frequency-domain descriptors, automatic feature selection, or even dimensionality reduction may help slice off redundancy and considerably reduce the computational load. Further, there is a prospect for application of the concept of Pose-LBP encoding to other classifiers or integration with deep learning models in further pursuit of performance. Finally, longitudinal studies connecting automated movement analysis with clinical diagnoses would help validate the practical value of such a system for early neurodevelopmental screening and decision support.

## Figures and Tables

**Table 1:** CSS-kNN-E performance under 10-fold cross-validation.

Fold	Accuracy	Sensitivity	Specificity	F1-Score
1	0.99101	0.99300	0.99739	0.99304
2	0.99102	0.99088	0.99754	0.99168
3	0.99641	0.99694	0.99891	0.99730
4	0.99461	0.99607	0.99844	0.99613
5	0.99461	0.99607	0.99848	0.99577
6	0.99102	0.99217	0.99746	0.99187
7	0.98384	0.98688	0.99560	0.98468
8	0.98381	0.98035	0.99518	0.98341
9	0.99101	0.99304	0.99744	0.99274
10	0.99820	0.99394	0.99939	0.99649
Mean ± Std	0.9916 ± 0.0046	0.9919 ± 0.0049	0.9976 ± 0.0013	0.9923 ± 0.0047

**Table 2:** Ablation study results of the CSS-KNN-E classifier (mean ± standard deviation) under 10-fold cross-validation.

#	Method/Configuration	Accuracy	Sensitivity	Specificity	F1-Score
1	Full (PoseHist + Motion) + Cost + Subspace kNN	0.9916 ± 0.0048	0.9919 ± 0.0050	0.9976 ± 0.0013	0.9923 ± 0.0048
2	No Cost (PoseHist + Motion) + Subspace kNN	0.9919 ± 0.0045	0.9922 ± 0.0044	0.9977 ± 0.0012	0.9926 ± 0.0045
3	PoseHist only + Cost + Subspace kNN	0.9777 ± 0.0072	0.9769 ± 0.0101	0.9937 ± 0.0021	0.9777 ± 0.0085
4	Motion-only + Cost + Subspace kNN	0.9373 ± 0.0136	0.9410 ± 0.0150	0.9826 ± 0.0038	0.9394 ± 0.0141
5	Single kNN (PoseHist + Motion) + Cost	0.9914 ± 0.0046	0.9918 ± 0.0049	0.9976 ± 0.0012	0.9921 ± 0.0049
6	Full model with Euclidean distance	0.9874 ± 0.0056	0.9873 ± 0.0078	0.9964 ± 0.0015	0.9878 ± 0.0068
7	Full model with k = 3	0.9867 ± 0.0068	0.9906 ± 0.0064	0.9966 ± 0.0018	0.9875 ± 0.0073
8	Full model with 100 ensemble cycles	0.9917 ± 0.0050	0.9920 ± 0.0050	0.9976 ± 0.0014	0.9924 ± 0.0049

**Table 3:** The performance evaluation of the various machine learning methods under 10-fold cross-validation.

Method	Accuracy	Sensitivity	Specificity	F1-Score
Decision Tree	0.6286 ± 0.0231	0.5838 ± 0.0340	0.8967 ± 0.0069	0.5854 ± 0.0304
SVM (Linear)	0.6105 ± 0.0221	0.5415 ± 0.0194	0.8845 ± 0.0062	0.5687 ± 0.0221
SVM (RBF)	0.4127 ± 0.0016	0.1992 ± 0.0006	0.7996 ± 0.0003	0.5843 ± 0.0016
Naive Bayes	0.2165 ± 0.0601	0.3561 ± 0.0591	0.8244 ± 0.0080	0.2133 ± 0.0643
Discriminant Analysis	0.5776 ± 0.0188	0.5221 ± 0.0148	0.8762 ± 0.0055	0.5421 ± 0.0181
Bagged Trees	0.8690 ± 0.0129	0.8068 ± 0.0176	0.9579 ± 0.0043	0.8528 ± 0.0139
AdaBoostM2	0.5702 ± 0.0194	0.4316 ± 0.0324	0.8660 ± 0.0067	0.4665 ± 0.0399
RUSBoost	0.4865 ± 0.0155	0.5260 ± 0.0214	0.8665 ± 0.0036	0.4563 ± 0.0190
Neural Network	0.7276 ± 0.0214	0.7052 ± 0.0276	0.9257 ± 0.0058	0.7014 ± 0.0276
1D-CNN	0.8834 ± 0.0382	0.8603 ± 0.0488	0.8659 ± 0.0538	0.8618 ± 0.0507
LSTM	0.9397 ± 0.0130	0.9386 ± 0.0119	0.9341 ± 0.0162	0.9369 ± 0.0146

**Table 4:** The performance evaluation of the RVI-38 and MINI-RGBD datasets.

Authors	Method	Dataset	Accuracy (%)
McCay et al. [34]	Pose and velocity, ensemble learning	RVI-38	97.37
		MINI-RGBD	100
Nguyen-Thai et al. [35]	A spatiotemporal attention-based model	RVI-38	81.58
		MINI-RGBD	91.67
Sakkos et al. [36]	Deep Neural Network	RVI-38	84.21
		MINI-RGBD	100
Adde et al. [37]	The variability of the centroid of motion	RVI-38	86.84
		MINI-RGBD	75.00
Proposed method	Pose-LBP + CSS-kNN ensemble	RVI-38	98.06
		MINI-RGBD	99.85

## Data Availability

The datasets generated and/or analyzed during the current study are not publicly available due to privacy and ethical restrictions involving human subjects, but are available from the corresponding author on reasonable request.
